# New Strategies to Kill Metabolically-Dormant Cells Directly Bypassing the Need for Active Cellular Processes

**DOI:** 10.3390/antibiotics12061044

**Published:** 2023-06-12

**Authors:** Karolina Stojowska-Swędrzyńska, Dorota Kuczyńska-Wiśnik, Ewa Laskowska

**Affiliations:** Department of General and Medical Biochemistry, Faculty of Biology, University of Gdansk, Wita Stwosza 59, 80-308 Gdansk, Poland; dorota.kuczynska-wisnik@ug.edu.pl (D.K.-W.); ewa.laskowska@ug.edu.pl (E.L.)

**Keywords:** antimicrobial peptides, biofilm, lysins, persister cells, phage-derived peptidoglycan hydrolases, polysaccharide depolymerases

## Abstract

Antibiotic therapy failure is often caused by the presence of persister cells, which are metabolically-dormant bacteria capable of surviving exposure to antimicrobials. Under favorable conditions, persisters can resume growth leading to recurrent infections. Moreover, several studies have indicated that persisters may promote the evolution of antimicrobial resistance and facilitate the selection of specific resistant mutants; therefore, in light of the increasing numbers of multidrug-resistant infections worldwide, developing efficient strategies against dormant cells is of paramount importance. In this review, we present and discuss the efficacy of various agents whose antimicrobial activity is independent of the metabolic status of the bacteria as they target cell envelope structures. Since the biofilm-environment is favorable for the formation of dormant subpopulations, anti-persister strategies should also include agents that destroy the biofilm matrix or inhibit biofilm development. This article reviews examples of selected cell wall hydrolases, polysaccharide depolymerases and antimicrobial peptides. Their combination with standard antibiotics seems to be the most promising approach in combating persistent infections.

## 1. Introduction

Increasing antimicrobial resistance (AMR) among pathogenic bacteria is one of the greatest public health threats. According to predictive models, in 2019, approximately 4.95 million deaths were related to AMR, with AMR being the direct cause of 1.27 million deaths [1]. Misuse and the overuse of antibiotics are the leading causes of the development of drug-resistant pathogens. Bacteria can acquire drug resistance by horizontal gene transfer or mutations; however, a growing body of evidence has demonstrated that antibiotic resistance can often be a transient and non-inherited phenomenon [2,3,4].

**Figure 1:**
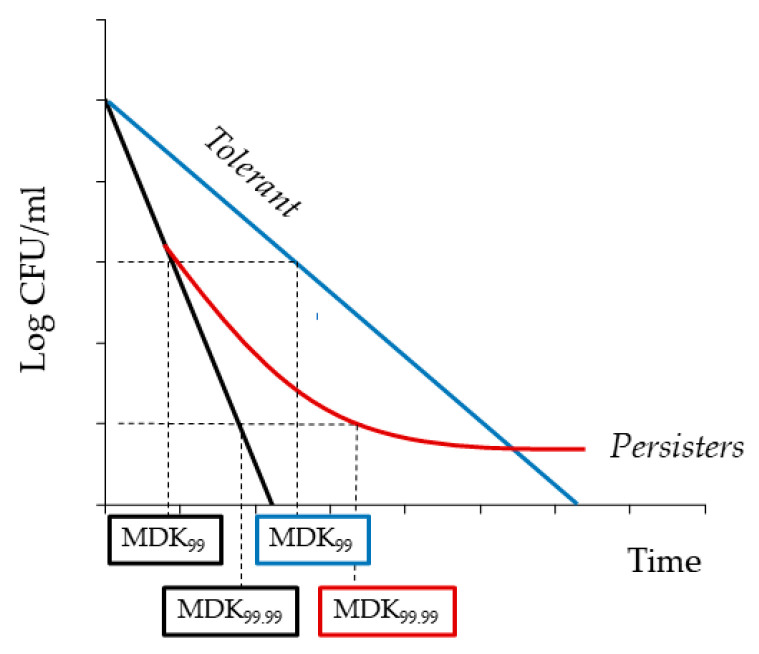
Time-kill curves of susceptible (black line), tolerant (blue line) and persistent (red line) populations. Bi-phasic killing curve typical for persistence is shown. All three strains have similar MIC values but differ in the minimum duration for the killing (MDK) of 99% or 99.99% of cells. The MDK99 for a tolerant strain and MDK99.99 for persistent bacteria are higher than the MDK99 and MDK99.99 for a susceptible strain, respectively.

An example is slow-growing or non-growing persister bacteria which can survive high concentrations of antibiotics, exciting even a 10-fold minimum inhibitory concentration [2,3,4]. Most currently used antibiotics target processes that occur only in growing cells (e.g., translation, replication, or peptidoglycan synthesis); hence, dormant or metabolically-inactive persisters become insusceptible to antibiotic action. Persisters, as phenotypic variants of wild-type genetically identical bacteria, can resume growth after removing the antibiotic, giving rise to drug-sensitive cells. A biphasic killing curve indicates the presence of persisters which usually constitute a small part of a population (0.001–1%) (Figure 1). In a culture exposed to an antibiotic, the fast killing of the majority of sensitive cells is followed by a slower and gradual decline of surviving bacteria in the culture. The term “persistence”, describing the behavior of a small subpopulation, should be distinguished from “tolerance”, which is an attribute of whole bacterial populations [3,4]. The MIC values for persisters and a tolerant population remain the same compared to a susceptible strain; however, the time required to eliminate the vast majority of cells (for example, the minimum duration for killing 99% or 99.99% of cells) is significantly extended (Figure 1). 

Much effort has been directed into identifying the trigger signals and genes responsible for persister formation. An equally important question is why only a limited number of cells become persistent, although the whole population is affected by a potential trigger signal. Ballaban et al. observed the growth of individual bacteria and found that persisters could be clearly distinguished from normal cells even before antibiotic treatment by their reduced growth rate [5]. Based on these observations, two ways by which cells can enter persistence have been proposed. Type I persisters require a triggering signal, for example, starvation during the stationary phase or other stressful conditions (i.e., the cells remain persistent even when the trigger conditions are removed), whereas type II persisters are continuously and stochastically generated during growth. The stochastic appearance of persisters can be explained by the fact that the amount of protein produced by a particular gene can vary from cell to cell leading to heterogeneous populations [6]. Recently, it has been demonstrated that fluctuations in the abundance of energy-generating Krebs cycle enzymes resulted in ATP deficiency and the stochastic formation of persisters [7]. Mathematical models imply that heterogeneous microbial populations demonstrate increased fitness compared to homogenous populations [8], and according to these models, forming persisters is a bet-hedging strategy to maximize the survival of heterogeneous populations under variable conditions [8].

Persistence (i.e., type I persisters) can be promoted by various stresses: starvation, oxidative stress, heat shock, acid or antibiotic treatment [9,10,11,12,13,14]. The accumulation of aggregated proteins has also been found to affect persister levels [15,16,17]. Numerous studies have demonstrated that the stringent response, SOS response, and toxin-antitoxin modules are implicated in the formation of a persister. The stringent response is mediated by an alarmone guanosine penta/tetraphosphate (pppGpp/ppGpp), the global regulator of metabolism. In response to starvation and other stresses, (p)ppGpp downregulates ribosomal RNA and ribosomal protein expressions via an interaction with its main target RNA polymerase [18]. Thus, changes evoked by (p)ppGpp signaling enable the transition of bacteria into a dormant state. The (p)ppGpp-dependent formation of persisters involves various mechanisms, including toxin-antitoxin modules. For example, it has been demonstrated that (p)ppGpp, in cooperation with the GTPase Obg, activates the transcription of the toxin HokB in *E. coli*. HokB, encoded by the *hokB-sokB* type I toxin-antitoxin module, forms pores in the membrane leading to membrane depolarization, ATP leakage, and persistence [18]. Another proposed mechanism relies on the (p)ppGpp-dependent inactivation and dimerization of *E. coli* ribosomes by the ribosome modulation factor RMF, the hibernation-promoting factor Hpf, and the ribosome-associated inhibitor RaiA [19]. It should be noted that there are also examples of persisters formed in a (p)ppGpp-independent way (as reviewed in [12]) or even persisters that originated from metabolically-active cells [20,21]. All these findings indicate that multiple mechanisms and pathways can be involved in persister formation. These mechanisms are described and discussed in recently published excellent reviews [2,12,22,23,24]. 

Numerous independent studies indicate that persisters constitute a heterogeneous group composed of different cells with varying metabolic statuses from “shallow” to “deep” persisters [25]. After removing the antibiotic, individual persisters resuscitate at different rates depending on their metabolic status. When stationary-phase bacteria are transferred to a fresh medium, “shallow” persisters wake up and become susceptible to antibiotics much earlier than “deep” persisters [26]. When extremally “deep” persisters remain dormant for a relatively long time, they become viable but not-culturable (VBNC) cells [27,28]. The existence of VBNC bacteria, which fail to grow on the routine bacteriological media, has been discussed for many years. One of the hypotheses suggested that VBNC bacteria are moribund or even dead cells in which a suicide program has been activated [29]; however, recent studies indicate that VBNC cells are able to restore their metabolic activity and resuscitate under favorable conditions. Several similarities between persisters and VBNC cells, including antibiotic tolerance, have been documented [28]. Some authors claim that both terms, namely, “persisters” and “VBNC”, describe the same phenotype [30]. Similarly to persisters, VBNC bacteria can appear stochastically or in response to certain stimuli. Moreover, the same conditions may trigger the formation of VBNC cells and persisters. Ayrapetyan et al. reported that both states, namely, VBNC and persistence, coexisted and were induced during the incubation of bacteria in human serum [27]. The duration of the lag phase before resuscitation is the main difference between persisters and VBNC cells. Pu et al. proposed that “dormancy depth” and the lag time before the re-growth of dormant *Escherichia coli* cells correlate with the level of protein aggregates that are formed due to the depletion of intracellular adenosine triphosphate (ATP) [17]. The removal of the “aggresomes” by the molecular chaperones DnaK-ClpB appeared to be a prerequisite for resuscitation [17]. Other studies demonstrated that the resuscitation rate might depend on the ribosome content. In heterogenous populations formed in response to antibiotics or heat shock, those dormant bacteria that had less ribosome regrowth were much slower than cells with a greater ribosome content [31,32].

**Figure 2:**
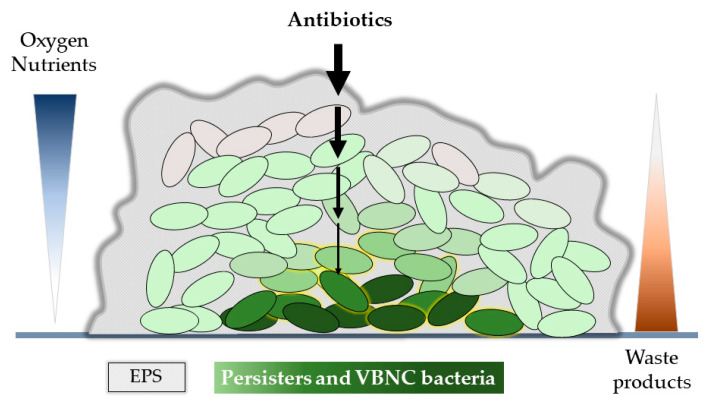
A heterogenous biofilm environment favors the formation of persisters and VBNC bacteria. In biofilms, bacteria are surrounded by extracellular polymeric substances (EPS), which form a barrier hindering the penetration of antibiotics. Due to the concentration gradients of oxygen, nutrients and waste products, bacteria are exposed to a different set of environmental conditions. In deeper biofilm layers, limited access to oxygen and nutrients slows down the metabolism and induces dormancy. See the text for more details.

The natural environment that favors the formation of persisters and VBNC bacteria is biofilm. Biofilms, as multicellular communities, can develop on biotic or abiotic surfaces and, according to the National Institutes of Health, are believed to be responsible for up to 80% of human bacterial infections [33]. Biofilm-forming bacteria are difficult to eradicate since they can be nearly 1000-fold more resistant to antimicrobial agents than their planktonic, free-floating counterparts [34]. Multiple factors may be responsible for biofilm drug tolerance and resistance (Figure 2). Extracellular polymeric substances (EPS), including polysaccharides, proteins, nucleic acids, lipids and other biopolymers, form a biofilm matrix, which can account for over 90% of the dry mass of the biofilm [35]. EPSs surround individual cells and microcolonies, acting as a scaffold and a milieu for the exchange of quorum sensing and other signals within the community. As a barrier, the matrix enhances tolerance to antimicrobials, immune cells and lethal environmental factors such as UV radiation, metal ions, extreme pH and desiccation [36,37]. The negatively-charged components of EPS, exopolysaccharides, or nucleic acids may interact and delay the penetration of positively-charged antibiotics. Slow diffusion and exposition to low doses of antibiotics enable bacteria to adapt to the drug by triggering stress responses and the acquisition of antibiotic tolerance. Furthermore, the specific microenvironment in deeper layers of the biofilm (e.g., a low pH, secondary metabolites, oxygen and nutrient limitation) may inactivate antibiotics and favor the formation of slow-growing and dormant cells [38,39,40].

One should keep in mind that bacterial persistence may promote the evolution of AMR. Especially, intermittent antibiotic treatments may lead to the selection of specific AMR mutations [41,42,43,44]; therefore, developing efficient strategies against dormant cells is of particular importance. Three main approaches have been intensively investigated so far: (1) resuscitation of dormant cells into metabolically-active states to sensitize them to conventional antibiotics; (2) interfering with persistence-related pathways (e.g., inhibitors of the stringent response, SOS response or quorum sensing); (3) direct killing of dormant bacteria by targeting cell envelope structures as membranes and peptidoglycan [2]. 

The last strategy is the main focus of this review. Targeting persister cells in both planktonic and biofilm populations is important for the effective treatment of bacterial infections. This review will provide a comprehensive overview of synthetic, phage and bacterial chemical molecules that disrupt the bacterial cell envelope, including the membrane(s), cell wall and biofilm matrix, and lead to the lysis of both wild-type and persister cells. 

## 2. Methods

A comprehensive literature search was conducted using the PubMed, Web of Science, and Google Scholar databases to identify relevant studies on agents specifically targeting bacteria, irrespective of their metabolic activity. The search was limited to articles published between 2010 and 2023, with a particular focus on the last decade. Various keywords such as “persister”, “biofilm”, “lysins”, “antimicrobial peptides”, “polysaccharide depolymerase”, and “eradication” were employed to identify the relevant articles. A total of 45 original research articles and 21 reviews were identified and selected based on their direct and documented connection with bacterial persistence or biofilm.

## 3. Bacterial Cell Envelope

**Figure 3:**
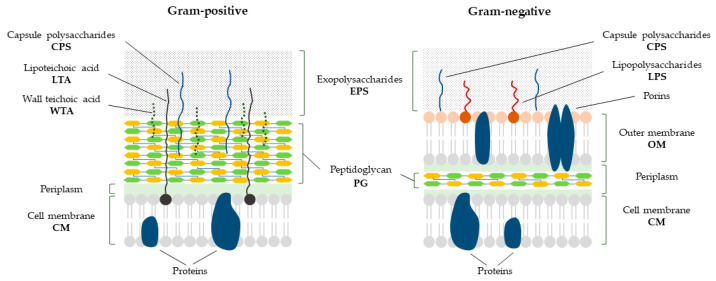
Gram-positive and Gram-negative bacterial envelope. See the text for more details.

The bacterial cell envelope is a sophisticated and intricate structure that functions as a shield to safeguard these organisms from their frequently inhospitable and unpredictable surroundings. It is a complex and dynamic structure that consists of several layers, including the cell membrane(s), cell wall, and sometimes an outer capsule or slime layer (Figure 3). The structure and composition of the bacterial cell envelope can vary depending on the type of bacteria and their environmental conditions [45,46]. It allows both the selective flow of nutrients from the outside and metabolic products from the inside [47].

The cytoplasmic or inner cell membrane (Figure 3, CM) is the innermost layer of the bacterial cell envelope and is composed of a phospholipid bilayer that acts as a selectively-permeable barrier to regulate the flow of substances in and out of the cell. The cell membrane also contains various proteins that perform essential functions such as the transport of nutrients and waste products, energy production, and signal transduction [45].

**Figure 4:**
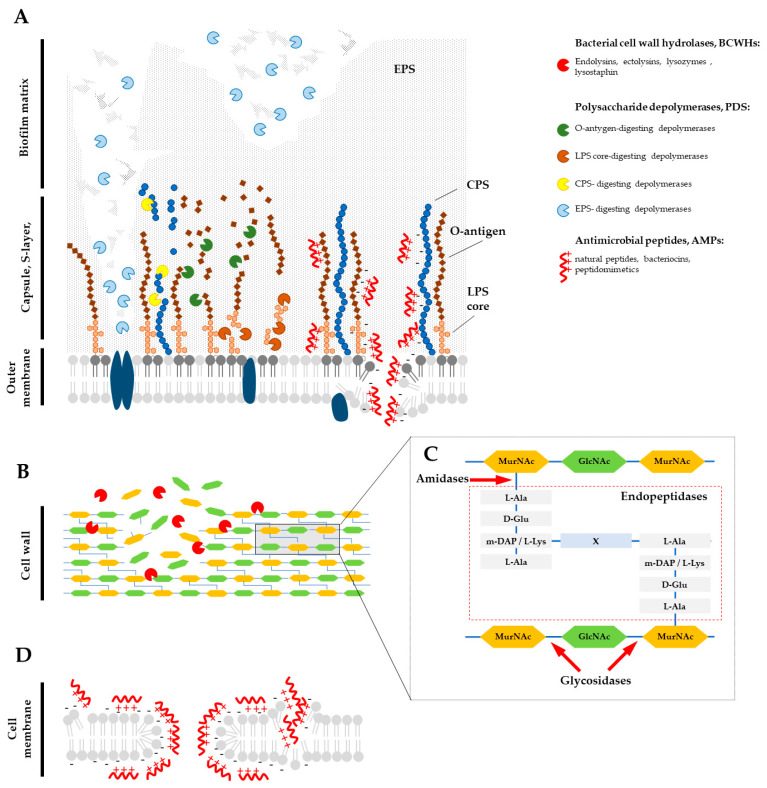
Targeting metabolically-inactive bacteria: molecular targets for bacterial killing. (**A**) The extracellular matrix of biofilms, along with the capsule layer and the S-layer, can be disrupted through mechanical, chemical, or enzymatic means using active molecules such as antimicrobial peptides (AMPs) or enzymes such as polysaccharide depolymerases (PSDs). PSD specifically digests saccharides found in the biofilm matrix, including cellular polymeric substances (EPS), and on the cell surface, such as lipopolysaccharides (LPS) and capsular polysaccharides (CPS), thereby targeting the structural integrity of the biofilm and the bacterial extracellular envelope. AMPs, as cationic peptides, interact with the negatively charged components of the biofilm, leading to the disruption of the biofilm matrix. This disruption results in the release of bacterial cells from the biofilm matrix and increases their susceptibility to other antimicrobial interventions. Bacterial membranes, such as the outer membrane in Gram-negative bacteria (**A**) and the cell membrane (**D**) in both Gram-negative and Gram-positive bacteria, can be effectively disrupted through the use of AMPs. These peptides possess a unique ability to interact with the lipid bilayer, leading to membrane destabilization, leakage of cellular contents, and eventual cell death. (**B**) The bacterial cell wall can be degraded through the action of bacterial cell wall hydrolases (BCWH). These hydrolases hydrolyze the bonds within the peptidoglycan structure, leading to significant cell wall degradation and subsequent cell lysis under osmotic pressure. (**C**) Bacterial peptidoglycan structure magnification. MurNAc: N-acetylmuramic acid; GlcNAc: N-acetylglucosamine; m-DAP: *meso-*diaminopimelic acid; X: the interpeptide bridge. Amidases and glycosidases can cleave the peptidoglycan structure at specific sites indicated by the red arrows. Endopeptidases cleave at various locations along the peptide bridges. These mechanisms of action are effective against both persister and non-persister cells, regardless of their metabolic activities.

The cell wall is the next layer surrounding the CM, providing structural support and protection to the bacterial cell. Comprised of peptidoglycan (Figure 3, PG), a complex polymer of sugars and amino acids, the cell wall provides resistance to osmotic pressure and physical stress. This distinctive and highly preserved layer is present in all bacterial cells. Its composition involves linear chains formed by alternating units of N-acetylmuramic acid (MurNAc) and N-acetylglucosamine (GlcNAc) residues [48]. These linear strands are cross-linked through a peptide side chain, which consists of a combination of L- and D-form amino acids (Figure 4C) [49,50]. The PG layer plays a crucial role in upholding the structural integrity and protection of bacterial cells by providing rigidity [51]. When it degrades, the cell loses its structural stability and becomes more prone to changes in external osmolarity, ultimately resulting in its collapse under osmotic pressures between the cell and its environment [51]; however, certain bacteria, referred to as cell wall-deficient bacteria (CWDB), have evolved specific adaptations to withstand osmotic pressure and to maintain viability in the absence of a cell wall [52,53] (discussed in more detail in Section 4.1.2). 

While the chemical structure of PG in Gram-positive and Gram-negative bacteria is similar, there are significant differences in the structure of their cell walls. Specifically, the thickness of the layers surrounding the CM differs between the two groups. In Gram-negative bacteria, the PG is only a few nanometers thick, typically consisting of one to a few layers. In contrast, Gram-positive bacteria have a much thicker PG, ranging from 30 to 100 nm in thickness and containing multiple layers. This difference in thickness can have important implications for the overall structure and properties of the bacterial cell wall [50].

The most notable variation in the structure of PG is the type of peptide crosslinks present between the glycan strands. The peptide bridges are typically composed of short peptide chains that are crosslinked by a single amino acid, such as lysine or alanine; however, the exact composition, length and arrangement of these peptide crosslinks (indicated as X in Figure 4C) can vary widely among different bacterial species, resulting in different peptidoglycan chemotypes [49,50]. The structural variations of PG in bacteria can have significant effects on the strength and stability of the bacterial cell wall. Additionally, changes in the composition or structure of PG can impact the activity of lytic enzymes that target the cell wall, as well as the ability of bacteria to resist host defenses and antibiotic treatments [49].

In the cell envelope of many Gram-positive bacteria, the teichoic acids, a family of negatively-charged polymers, are present [54]. They are composed of glycerol or ribitol phosphate chains that are attached to the PG of the cell wall. Teichoic acids are classified into two main types: wall teichoic acids (Figure 3, WTA) and lipoteichoic acids (Figure 3, LTA) [55]. WTAs are covalently linked to the PG layer and can extend several nanometers into the extracellular space. They play a key role in maintaining the structural integrity of the cell wall and contribute to its negative charge, which is important for ion exchange and cell surface interactions. In addition, WTAs are involved in bacterial adhesion to host cells and in the regulation of autolysin activity, which is required for cell growth and division [56,57]. LTAs are anchored to the CM through a glycolipid moiety and can extend through the PG layer into the extracellular space. They are involved in a variety of cellular functions, including cell division, cell wall metabolism, and cell signaling. LTAs can also stimulate the host immune system and contribute to the pathogenicity of some bacterial species. Moreover, teichoic acids can play a role in the degradation of the cell walls by lysins. The negative charge of teichoic acids can interact with the positively-charged lysins, aiding their binding to the cell wall and facilitating their enzymatic activity [55,58]. 

Since Gram-negative bacteria have a thin PG layer compared to Gram-positive bacteria, they have evolved a unique structure known as the outer membrane (Figure 3, OM). This OM lies outside of the PG layer, provides additional protection to the cell and helps to maintain its structural integrity. The composition of OM may vary depending on the species and environmental conditions. The OM is mainly composed of lipopolysaccharides (Figure 3, LPS) and phospholipids [59], which act as a selective permeability barrier, control the transport of molecules into and out of the cell and prevent the entry of large molecules, such as lysins and other bacterial wall hydrolases, into the periplasmic space where the bacterial cell wall resides [60].

Many bacterial cells are surrounded by a layer of extracellular polymeric substances (Figure 3, EPS), mostly exopolysaccharides, that are either covalently bound to the cell surface or excreted as a loose slime and form a capsule or slime layer (S-layer), respectively [61]. The capsule and S-layer can serve various functions depending on the species and the environmental conditions. A capsule is a dense, tightly packed, up to 10 µm thick layer of capsular polysaccharides (Figure 3, CPS) that surrounds the cell. Capsules can provide protection from host immune responses and can also help the cell to adhere to surfaces or other cells. The slime layer is a looser, more diffuse, amorphous layer of polysaccharides that can protect the cell from environmental stresses such as desiccation or osmotic shock [62]. 

EPSs and CPSs are both involved in the formation of the biofilm matrix, which they help to maintain by holding bacterial cells together and allowing the biofilm to adhere to surfaces [35,63]. The composition of the polysaccharides in EPS and CPS can vary depending on the bacterial species or strain that forms the biofilm, as well as whether the biofilm consists of single or multiple species. It is important to note that multi-species biofilms are more common in nature and can contain a diverse range of organisms, such as bacteria, algae and fungi [35], further expanding the composition of the biofilm matrix [64].

Moreover, the composition of the EPSs matrix within individual biofilms may vary depending on a number of factors such as the type of nutrients, the prevailing environmental conditions, the stage of growth and the fitness of the host cells [65]. As mentioned before (Figure 2), the matrix restricts the diffusion of antimicrobial agents and other chemicals into the biofilm; therefore, disrupting the biofilm structure, whether mechanically or chemically, is a crucial stage in the treatment of bacterial infections. This disruption can enhance the effectiveness of antimicrobial agents and make them more accessible to the bacteria. Figure 4 summarizes agents that combat the bacterial cell envelope and cause the destruction of the biofilm matrix [48,49,66,67,68]. Detailed mechanisms of these agents’ activities are presented in the following sections.

## 4. Bacterial Cell Wall Hydrolases

Bacterial cell wall hydrolases (BCWH) play a crucial role in degrading PG. They exhibit rapid bactericidal activity, enabling them to swiftly kill bacteria upon contact [69], regardless of their metabolic activity, resulting in bacteriolysis. Their enzymatic activity is exerted through the hydrolysis of the glycosidic bonds between MurNAc and GlcNAc in the polysaccharide backbone, as well as the amide and peptide bonds in the interpeptide bridge of peptidoglycan (Figure 4C). BCWHs can be utilized in various forms for the treatment of infectious diseases. These include purified native enzymes, denatured enzymes, partial digests [70] and recombinant proteins expressed in transgenic animals or plants to enhance host defense [71]. BCWHs can be derived from diverse sources such as animals (e.g., lysozymes), insects, plants, bacteria (e.g., lysostaphin), and phages (e.g., lysins) [72,73,74,75].

Phage-derived lysins have shown the greatest potential in killing persistent cells and destroying biofilms. They exhibit remarkable specificity towards their target bacteria, having evolved to recognize and specifically bind to certain cell wall components. This specificity is crucial for their effectiveness in combating bacterial infections as it enables them to precisely target the bacteria of interest while minimizing any potential impact on beneficial bacteria or host cells. On the other hand, bacterial and eukaryotic lysins are less specific than phage lysins. They exhibit activity against a broader spectrum of microorganisms, which can be both advantageous and disadvantageous. While they may have a wider range of targets, their non-specificity can also lead to unintended effects on the commensal bacterial flora of the host [76]. 

Furthermore, bacterial and eukaryotic lysins may possess certain features that limit or reduce their effectiveness against persistent bacteria. These features can include mechanisms of resistance developed by bacteria to evade the action of lysins or the inability of lysins to penetrate biofilms effectively and reach the target cells. For example, lysozyme exhibits both enzymatic (lytic) and non-enzymatic (on-lytic) activity [76]. Its primary antibacterial action is directed towards Gram-positive bacteria, where the enzymatic activity on PG is most effective. In the case of Gram-negative bacteria, the presence of a protective OM significantly reduces the effectiveness of lysozyme [77,78]. Unfortunately, some Gram-positive bacteria have developed defense mechanisms against lysozyme action due to the production of chemical peptidoglycan variants that preclude lysozyme from binding to it [79]. Crucially, the addition of exogenous lysozyme allows the bacteria to escape into the L-form, thus protecting against cell killing by active cell wall antibiotics [80]. The on-lytic activity of lysozyme is attributed to its cationic and hydrophobic properties. It possesses the ability to insert into and create pores within negatively-charged bacterial membranes [81,82]. By doing so, lysozyme causes damage to the cell wall by activating a bacterial autolytic wall muramidase, a phenomenon known as the “Trojan horse” effect, ultimately leading to bacterial autolysis [83]. Since autolysis refers to the active breakdown of cellular components leading to cell death, it is unattainable for metabolically-inactive survival cells. Lysozyme’s effectiveness due to its cationic properties is primarily attributed to its ability to prevent biofilm formation with the combination of antibiotics, disinfectants and AMPs [76]. 

### 4.1. Phage-Derived Peptidoglycan Hydrolases

Phage-encoded peptidoglycan hydrolases (PGH), also known as lysins, play an essential role in the lytic cycle of tailed phages. There are two types of lysins involved: (i) ectolysins known as virion-associated lysins (VAL) or virion-associated peptidoglycan hydrolases (VAPGH), which facilitate the entry of viral DNA into the host cell by promoting the local degradation of peptidoglycan from the outside [84,85]; and (ii) endolysins, which degrade peptidoglycan from within the host cell, causing it to burst at the end of the reproductive cycle. This process facilitates the release of phage progeny [86,87,88]. Both types of lysins, when expressed recombinantly and applied externally, can cause lysis-from-without and kill bacteria due to turgor pressure [89]. PGHs show a high degree of diversity in their structures, which affects their catalytic activity and specificity [90]. Enzymes specifically targeting Gram-positive bacteria possess a modular domain structure comprising two main components: an N-terminal enzymatically active domain (EAD) and a C-terminal cell wall binding domain (CBD), connected by a flexible linker region [66,90]. Enzymes that target Gram-negative bacteria are generally globular and lack CBDs [90]. The CBD domain is responsible for recognizing and binding to the bacterial cell wall and has a high affinity and specificity for the receptors on the surface of the target bacteria. Its presence is originally attributed to its role in binding endolysins released during the lytic cycle to cellular debris, thereby preventing the lysis of uninfected cells [91]. This feature is crucial in lysins targeting Gram-positive bacteria; however, in lysins targeting Gram-negative bacteria, the CBD domain is unnecessary due to the additional OM layer that protects uninfected bacteria. The EADs are responsible for specifically degrading peptidoglycan (PG). The catalytic activities of PGHs can be further classified into glycosidases (including lysozyme, transglycosylases, and glucosaminidases), amidases, and endopeptidases [66,87] (Figure 4C).

The combined action of the EAD and CBD determines the specificity of the lysin. This specificity can vary, limiting its antibacterial effect to specific classifications such as genus, species, or even certain strains [87,88,91,92,93,94]. In rare cases, lysins may have a broad spectrum of activity. For example, the PlyC endolysin targets several *streptococcal* species [95], the PlySs2 endolysin has lytic activity against methicillin-resistant *Staphylococcus aureus* (MRSA) [96] and Art-175 endolysin has bactericidal activity not only against the *Pseudomonadaceae* family but also against some *Enterobacteriaceae* members [97].

The near-species specificity of lysins allows for the selective killing of target pathogens, avoiding unnecessary harm to non-targeted beneficial bacteria [98,99]. PGHs are not known to have any off-target effects. This means that they are unlikely to harm other cells in the body and can be used as targeted therapies. This is a significant advantage over broad-spectrum antibiotics, which can disrupt the entire microbial community and lead to the development of antibiotic-resistant strains. PGHs are generally well-tolerated by the host organism, as host proteases rapidly degrade them, do not show cytotoxicity to mammalian cells [92,100] and do not significantly impact host microbiota [86].

PGHs, in their native form, have been successfully used to kill Gram-positive pathogens, such as MRSA [101,102,103,104], *Staphylococcus epidermidis* [105], vancomycin-resistant *Enterococcus faecalis* [106] and *Enterococcus faecium* [107], *Bacillus cereus* [108], *Clostridium* spp. [109] and many others [99].

It is worth noting that for Gram-positive cells, the PG layer is primarily accessible from the outside, which enables both endolysins and ectolysins to attack the bacterial cell from that direction; however, in Gram-negative cells, the presence of the OM creates a significant barrier that makes it more challenging for hydrophilic enzymes to penetrate and reach their target within the PG layer [60,110]. To fully lyse Gram-negative bacteria, PGHs must first penetrate and disrupt the OM. Due to this barrier, only a limited number of lysins have been found to have a natural intrinsic activity against Gram-negative bacteria [60,111]. To overcome the limitation posed by the OM in Gram-negative bacteria, researchers have focused on designing new recombinant lysins that are fused with OM-permeabilizing agents. These agents can include substances such as ethylenediaminetetraacetic acid (EDTA) [112], spanin complexes [113,114], or peptides that possess highly cationic properties [60]. When fused with lysins, these agents interact with the negatively charged components of the OM, leading to destabilization and permeabilization of the membrane. This allows the lysin to gain access to the peptidoglycan layer and exert its lethal activity [60]. One particularly effective approach is using the artilysin series, designed by Briers et al. [115,116]. Among them, Art-175 has demonstrated high bactericidal activity against various Gram-negative pathogens, including multidrug-resistant strains of *Pseudomonas aeruginosa* [97], *Acinetobacter baumannii* [69], *E. coli* [100], *Klebsiella pneumoniae* [97] and *Salmonella enterica* [97].

PGHs kill target pathogens regardless of their origin or level of multidrug resistance. For example, P128 exhibits equal effectiveness against both drug-sensitive and drug-resistant Coagulase-negative staphylococci (CoNS) strains, as evidenced by comparable MIC_50_ and MIC_90_ values [117]. Similarly, Art-175 demonstrates strong inhibitory effects on all tested strains of *P. aeruginosa* with no observed variations in susceptibility among the environmental, clinical and multidrug-resistant strains [97].

No persistent bacteria were found after treatment with lysins, indicating a high efficacy in eliminating bacterial cells. Although some bacterial cells may regrow after the first dose of lysin, they remain sensitive and are ultimately killed upon subsequent doses [69,97,105,117]. Lysins have shown remarkable efficacy in eliminating bacterial cells without promoting the emergence of resistant strains [118,119,120]. This is an important advantage in the context of antimicrobial therapy, as the emergence of resistant strains can undermine treatment effectiveness. The biodegradable nature of lysins further contributes to their low environmental impact. Lysins are expected to degrade naturally in the environment, which reduces the likelihood of resistance development and minimizes their persistence [86]. The low propensity for resistance development can be attributed to several factors. Firstly, lysins specifically target the PG layer of bacterial cell walls, which is essential for bacterial survival. Modifying the PG to evade lysin recognition or activity is challenging for bacteria, as any alterations are likely to have significant consequences for cell viability. Changes induced by lysins in the PG structure typically lead to detrimental effects on the bacterial cell, resulting in lysis and cell death [49,121].

Lysins exhibit rapid and highly specific action, allowing for the swift and efficient killing of bacterial cells. Unlike antibiotics, which may take hours to days to show an effect, lysins can act within minutes to hours [88]. The rapid bactericidal effect of Art-175 lysin has been observed through time-lapse microscopy, allowing the real-time visualization of bacterial cells and their response to lysin treatment. The findings indicate that upon encountering Art-175, bacterial cells are swiftly eradicated within seconds [69]. Osmotic lysis occurs immediately or after extensive degradation of the peptidoglycan layer, depending on the medium’s osmolarity and the consequent destabilization of cells due to internal osmotic pressure. This rapid action underscores the potent bactericidal activity of lysins and their ability to promptly eliminate bacterial pathogens upon exposure [69].

#### 4.1.1. Efficacy of Phage-Derived PGHs against Persistent Bacteria and Biofilm

The effectiveness of lysins against persistent bacteria has been directly examined in a limited number of studies. These particular lysins are listed in Table 1, summarizing their reported activities and characteristics. In these studies, persister cells were selected using different methods such as prior exposure to standard-of-care (SoC) antibiotics or induction with bacteriostatic agents such as rifampicin or ciprofloxacin [14,122]. Subsequently, the persister cells were treated with specific lysins. The results consistently demonstrated that lysins were highly effective in killing the persister bacteria, exhibiting comparable activity to that observed against actively growing cells.

In addition to their efficacy against persistent bacteria, lysins have been extensively studied under various growth conditions. These studies have investigated the activity of lysins against bacteria in different states, including slow-growing or non-growing conditions such as in saline or buffers, as well as during a stationary phase or starvation, which can induce different levels of dormancy, including persistence [119,120,123]. For example, Poonacha et al. demonstrated that treating CoNS cells with P128 lysin at 1xMIC resulted in a significant CFU reduction in less than 1 h, regardless of the medium used. The reduction in cell viability was observed in both lactated Ringer’s solution (LRS), which induces low metabolic activity and dormancy, and cation-adjusted Mueller–Hinton broth (CAMHB), which promotes a state of high metabolic activity [117]. The killing efficiency of bacteria in buffer solutions where the cells are physiologically dormant and have very low metabolic activity is the same as in complete bacterial culture media during intensive growth [117]; therefore, the effectiveness of lysine is not affected by the metabolic activity of the target bacteria. These findings highlight the robust and consistent activity of lysins against bacteria under different growth conditions, supporting their potential as effective antimicrobial agents for combating persistent bacterial infections.

Certain lysins have demonstrated their potential as antibiofilm agents [127,128] due to their ability to penetrate the matrix structure of biofilms and directly target the bacterial cells within the biofilm [105,127,128]. The bacteria can be entirely eliminated even if the matrix is only partially disrupted and the anti-biofilm activity of lysins is primarily due to their ability to kill the bacteria that are trapped in the biofilm matrix [129]. This leads to a reduction in the amount of biomass present in the biofilm and ultimately disrupts the biofilm structure. While some studies have suggested that lysins may also directly affect the biofilm matrix, the available evidence demonstrates that their primary mode of action is through killing the bacteria rather than any direct activity of lysins on the biofilm matrix itself [130].

Gutierrez et al. reported that LysH5 exhibited significant disrupting activity against biofilms formed by both *S. aureus* and *S. epidermidis* strains [105]. LysH5 removed staphylococcal biofilms irrespective of the extracellular matrix composition (e.g., polysaccharides, DNA, or proteins). The viable cells that survived after a single exposure to LysH5 did not show an enhanced capacity to form a biofilm. Moreover, it was observed that the surviving cells remained susceptible to the activity of the endolysin. Subsequent treatment of the biofilm cells that had survived the initial dose with LysH5 resulted in the effective eradication of the population, reducing it to undetectable levels. It was also noted that in certain strains, sub-MIC concentrations of LysH5 not only failed to induce the formation of *staphylococcal* biofilms but also exhibited a preventive effect [105].

Lysins can be used in combination with other therapies to enhance their bactericidal activity or prevent the emergence of resistance. For example, combining endolysins with antibiotics can effectively treat bacterial infections and reinfections, particularly those caused by antibiotic-resistant and dormant bacteria [131]. Letrado et al. [132] showed that treatment with Cpl-711 endolysin in combination with the antibiotic amoxicillin or cefotaxime was able to completely eradicate multidrug-resistant *S. pneumoniae* biofilms in vitro, which are notoriously difficult to treat with antibiotics alone [132]. The researchers suggested that the synergistic effect of Cpl-711 and amoxicillin may be due to the fact that Cpl-711 breaks down the PG layer, making it easier for the antibiotic to penetrate and kill the bacterial cells [132].

Combinations of P128 with SoC antibiotics such as daptomycin, vancomycin, and linezolid led to a decrease in the minimum inhibitory concentration (MIC) compared to the individual MICs of P128 or the antibiotics alone. The most pronounced synergy was observed when combining P128 with daptomycin against *S. lugdunensis* B9510, resulting in a 48-fold lower MIC for P128 and a 6-fold lower MIC for daptomycin [117]. The synergistic effect of P128 on both the planktonic cells and biofilms of CoNS isolates was observed across different antibiotic classes, indicating that the main mechanism underlying the synergy between P128 and antibiotics may involve the enhanced intracellular accumulation of antibiotics due to an increased permeability loss in P128-treated CoNS cells [117].

In 2010, Daniel et al. [133] conducted a study to evaluate the effectiveness of combining a phage-derived lysin called ClyS with the traditional antibiotic, oxacillin, to treat severe staphylococcal infections. The researchers found that when administered separately, neither ClyS (at a dose of 166 μg/mouse) nor oxacillin (at a dose of 100 μg/mouse) significantly improved the survival rate of mice compared to the control group that received only the buffer solution. The survival rates in these individual treatment groups ranged from 30% (6/20) to 35% (8/23). In contrast, the study demonstrated that a single dose of the combined treatment, where ClyS was administered intraperitoneally and oxacillin was administered intramuscularly at doses of either 50 or 100 μg, led to a significant increase in mouse survival rates. The survival rates in these combined treatment groups were 80% (8/10) and 82% (18/22), respectively [133].

The development of lysins targeting Gram-positive bacteria has proven to be a successful strategy in the development of novel antibacterial agents. Several lysins are currently in different phases of clinical trials, demonstrating their potential as effective therapeutics [99]. This success highlights the potential of lysins as a promising alternative to conventional antibiotics, which are increasingly ineffective against drug-resistant bacteria.

Lysins have demonstrated great potential in combating drug-resistant Gram-negative bacterial infections; however, several challenges need to be addressed before their widespread use is implemented in clinical settings. Some of these challenges include safety concerns with the use of OM permeabilizers such as EDTA, a limited efficacy against stationary-phase bacteria, and issues with enzyme stability. To maximize the therapeutic potential of lysins, scientists are exploring innovative approaches such as protein engineering and formulation sciences. By employing these strategies, they aim to improve the stability of lysins, thus ensuring their activity and effectiveness over extended periods of time. Furthermore, combination therapy involving lysins and other classes of antibacterial agents is being investigated as a promising solution. This approach leverages the synergistic effects of different antibacterial agents to overcome the limitations of lysins when used alone. By combining lysins with other drugs, researchers hope to enhance their overall efficacy and broaden their spectrum of activity against drug-resistant Gram-negative and Gram-positive bacterial infections [60].

#### 4.1.2. Lack of Effectiveness of Phage-Derived PGHs against Cell Wall-Deficient Bacteria

Loss of the cell wall does not necessarily kill the cell. It has been observed that cell wall-active triggers, such as certain antibiotics (e.g., penicillin or vancomycin) or lytic enzymes (e.g., lysozyme), can cause many bacteria to temporarily enter a state where they lack a cell wall [80,134,135,136]. In this wall-deficient state, CWDB bacteria such as L-forms and spheroplasts, have adaptations that allow them to withstand osmotic pressure and maintain their cellular integrity in the absence of a rigid cell wall. They may have altered membrane compositions or increased membrane fluidity to compensate for the lack of structural support provided by the cell wall [13]. Because CWDB forms lack a cell wall and the associated molecules, they inherently exhibit a resistance to compounds that target the PG; hence, the lack of a cell wall can be viewed as the “Achilles’ heel” for lysins, as it hinders their effectiveness against such bacteria [137].

CWDBs serve as persister cells with a remarkable ability to transform into non-walled cells when faced with antibiotic attacks targeting the cell wall. This transformation renders them tolerant to these drugs [138]. CWDBs can survive antibiotic therapy within a culture medium if they are in appropriate isotonic environment and can subsequently resume division as walled cells once the drugs are removed [139,140,141]. Unlike dormant persister cells, spheroplasts and L-forms maintain an active metabolism, indicating their continuous metabolic activity. Moreover, L-form cells have the ability to proliferate through an FtsZ-independent mechanism [134], where instead they employ an excessive production of membranes, resulting in the formation of internal or external vesicles. These vesicles serve as viable progeny, allowing L-form cells to propagate and maintain their populations [52,135]. The transformation into the L-form requires the presence of an osmoprotective medium and can occur in both Gram-positive and Gram-negative bacteria. In contrast, spheroplasts only occur in Gram-negative bacteria, can survive in media without osmotic protection, and do not exhibit cell proliferation [13,135].

In 2021, Ongenae et al. demonstrated that phage infection and the subsequent lysis of bacterial cells can induce the conversion of bacteria into L-forms within a population [139]. Notably, it was found that the addition of spent media remaining from cultures after phage exposure to bacteria that grow in osmoprotective media, resulted in the formation of CWDB. This confirms that the L-form conversion process is facilitated by the activity of endolysins, which are released after previous phage infection. The same results were observed for both *B. subtilis* as Gram-positive and *E. coli* as Gram-negative bacteria [139].

Wohlfarth et al. further supported this mechanism by confirming that the L-form conversion can occur through collateral damage caused by soluble phage endolysins released during repeated cycles of phage infection [137]. When sub-inhibitory doses of phages infect and lyse bacteria, the endolysins act as a switch, triggering the induction of L-forms. This leads to the transient persistence and preservation of bacterial community viability by facilitating the conversion of uninfected cells into L-forms prior to phage infection [137]. In consequence, the loss of the cell wall in L-forms and spheroplasts provides transient resistance against viral infections and external action of cell wall-targeting enzymes. This adaptation seems crucial for the survival of CWDB, which has been associated with various recurrent infectious diseases [142,143]. Antibiotic-tolerant CWDB have been observed in several bacterial pathogens, including *A. baumannii*, *K. pneumoniae*, *Enterobacter cloacae*, and *P. aeruginosa* [141,144,145,146]. The presence of CWDB in persistent and chronic infections highlights the need for novel therapeutic approaches to effectively eliminate these types of survival cells [138].

While the impact and roles of L-forms in the environment are not fully understood, they have been found in various natural sources, including plants, animals, and humans [138,147]. Evidence suggests bacterial wall deficiency may occur within infected eukaryotic cells [80,148]. Interestingly, L-forms are frequently detected in the clinical urine samples of elderly patients [143]. The L-form escape triggered by endolysins, and subsequent reversion could have important implications for future endolysin-based therapeutic interventions. L-forms and spheroplasts, lacking a cell wall but retaining an intact cell membrane, can be effectively targeted and killed using membrane-active agents such as AMPs. These AMPs interact with the bacterial cell membrane, leading to its destabilization, the leakage of cellular contents, and ultimately cell death [149] (as discussed in more detail in Section 6). Combination therapies involving non-cell wall-targeting drugs and antibiotics can be explored as new strategies to target different aspects of bacterial survival. One potentially promising approach is the combination of cell membrane targeting agents with phage-encoded lysins. This synergistic combination has the potential to enhance the effectiveness of bacterial killing significantly. By combining agents, the treatment can target multiple critical components of the bacterial cell, thereby increasing the overall efficacy in killing bacteria. This combination approach takes advantage of the complementary mechanisms of the action of the agents, potentially leading to a more potent and comprehensive antibacterial effect [113].

## 5. Phage-Derived Polysaccharide Depolymerases

Phages, during their life cycle, encode enzymes called polysaccharide depolymerases (PSD), which have an important role in recognizing, binding, and digesting the carbohydrate-based components of the bacterial cell envelope, including CPSs, LPSs O-polysaccharides and EPSs [67]. By degrading these components, PSDs can facilitate the attachment and entry of phages into bacterial cells. The PSDs enable phages to access the compatible adsorption receptors on the bacterial cell envelope surface, which are necessary for phage attachment and infection [150]. Some PSDs are also capable of degrading polypeptides or lipids, which may aid in further penetration of the bacterial cell wall or in the release of progeny virions during the lytic cycle of phage infection [151]. The PSDs can also degrade extracellular polysaccharides that surround bacterial biofilms. By degrading the EPS matrix, phages can penetrate and disintegrate the biofilm, making the bacterial cells more vulnerable to viral attack [152].

In nature, PSDs can be found associated with different components of the virion involved in virus invasion, as well as being released as free enzymes [153]. Through genetic engineering, these enzymes can be obtained and used externally, both in vitro and in vivo, to efficiently break down polysaccharides on the surface of bacterial cells and within the biofilm matrix [153].

PSDs exhibit a wide range of substrate specificities, providing a diverse set of functions to a narrow range of target polysaccharides [67,84]. Their high specificity limits their ability to digest certain types of polysaccharides and even depolymerases derived from closely related phages may not recognize the cell-surface polysaccharides produced by the same bacteria under different conditions [154]. To address the complexity of multi-species biofilms that exhibit a wide variety in their matrices, cocktails of different depolymerases can be used to target different agents within a biofilm [155].

The diversity of PSDs allows them to target a wide range of bacterial species and strains, making them potentially effective in treating persistent bacterial infections. Similarly to lysins, depolymerases can exhibit host specificity, leaving commensal flora unharmed [156]. The low risk of resistance to PSDs makes them an attractive option for combination therapies with antibiotics and other treatment approaches. Bacterial resistance to PSDs can evolve through modifications in the composition of bacterial polysaccharides, particularly in LPS or capsules. These modifications may result in defects in LPS or capsule structures, which can lead to reduced bacterial fitness or virulence [155,157]. It is important to note that modifications in bacterial polysaccharides can also result from other evolutionary pressures, such as bacterial adaptation to environmental stresses or host immune responses; therefore, the evolution of bacterial resistance to depolymerases is a complex process that involves multiple factors and may not always lead to a loss in bacterial fitness or virulence [153].

### Efficacy of Phage-Derived Polysaccharide Depolymerases against Persistent Bacteria and Biofilm

PSDs do not exhibit direct bacteriolytic or bacteriostatic activity. Instead, the therapeutic benefits of PSDs often arise from their ability to “strip away” extracellular polysaccharides that are used by bacterial pathogens to promote biofilm formation, virulence, and a defense against host immune responses, antimicrobial agents, and phages. By specifically degrading the protective polysaccharide layers surrounding bacterial cells, PSDs can help to disrupt the biofilm matrix and make the bacteria more vulnerable to immune responses [155,158] and antimicrobial treatments [159]. Studies have shown that PSDs can lead to a reduction in the biomass of pre-formed biofilms. Gutiérrez et al. showed that the EPS depolymerase Dpo7, used against staphylococcal biofilms, resulted in the reduction of biofilm biomass of *S. epidermidis* strains from 31% to 75% [160]. Another EPS depolymerase, Dpo1, led to a 20% reduction of *A. baumannii* biofilm [161,162,163]. The depolymerase Dpo42, which infects clinical isolates of *E. coli*, has the ability to decrease the biomass of preformed biofilms and prevent the formation of new bacterial biofilms [161].

However, it should be noted that complete biofilm removal or a complete inhibition of biofilm formation was not observed. On the contrary, Wu et al. provided evidence that the activity of depolymerase did not result in a significant reduction in the number of viable cells; however, they demonstrated that the depolymerase’s ability to degrade the extracellular material of the biofilm had a considerable impact. This degradation process facilitated the release of attached cells, thereby causing them to disperse in the solution. As a consequence, the overall biomass of the biofilms was reduced [164].

Phage-derived depolymerases are insufficient for the complete eradication of bacterial biofilms and the elimination of pathogenic bacteria; therefore, a combination therapy approach involving the simultaneous or sequential use of multiple agents is recommended. This approach may include antibiotics, phages, chemicals such as xylitol [165], or natural compounds (known to enhance antibiofilm activity of *Escherichia coli* CECT 434 [166]), as well as detergents such as chlorine dioxide [167] or disinfectants [168], in addition to phage-derived depolymerases. The combination of these agents aims to enhance the effectiveness of treatments against biofilm-related infections [75].

The rationale behind this approach is that using multiple agents that target different aspects of the biofilm can increase the overall effectiveness of the treatment compared to using a single agent alone. For example, combining a depolymerase Dep42 with antibiotic polymyxin can help to disrupt the biofilm matrix and increase the susceptibility of bacteria to the antibiotic, resulting in a higher reduction in the numbers of *K. pneumoniae* strain 2226 in biofilm [164]. Alternatively, using a phage combined with a depolymerase can help degrade the biofilm matrix and target the bacterial cells directly, e.g., the combination of KP34p57 depolymerase and depolymerase-non-bearing phage KP15 resulted in a higher reduction in the colony count of *K. pneumoniae* strain 77 [169]. Examples of combined therapy against bacterial biofilm formation are well-reviewed by Topka-Bielecka et al. [159].

The most promising and most effective are combinations of phage-derived depolymerases with BCWH or phages with bacterial cell wall lytic activity. Pertics et al. demonstrated that the combination of bacteriophage B1 and depolymerase K2 (B1dep) [170] leads to cell lysis of the hypervirulent strain *K. pneumoniae* 52145, which was initially resistant to phage 731 alone [171]. The B1dep depolymerase specifically degrades the K2 capsule enabling the B1 phage to degrade peptidoglycan and induce cell lysis. Other studies showed that the combination of poly-N-acetylglucosamine depolymerase (DA7) with phage-encoded hydrolase (LysK) had been proven to be highly effective against *S. aureus* biofilms and persisters [172]. DA7 does not have a bactericidal effect, but it leads to the degradation and destabilization of the three-dimensional structure of the biofilm and allows LysK to penetrate the deeper layers of the biofilm and to act directly on bacterial cells, leading to their lysis and death [172].

PSDs can be used as antimicrobials to break down bacterial polysaccharides, potentially making bacteria vulnerable to antibiotics or the immune system [153], including serum killing as well as phagocytosis. Studies have shown that phage-derived recombinant and purified PSDs effectively reduce bacterial virulence and promote immune-mediated killing, with high animal survival rates and no observed toxicity [75]. These PSDs have also been found to lower the levels of proinflammatory cytokines [173]. Similarly to other non-endogenous biologics such as therapeutic proteins, PSDs have the potential to cause allergic responses; however, no cases of allergic reactions to PSDs have been reported thus far [174], suggesting that they may be a safe and effective antimicrobial option.

Based on the available evidence, it can be predicted that phage-derived PSDs (i.e., phage-encoded depolymerases) hold great promise as a novel class of antimicrobials; however, their full potential can be realized when used in combination with other agents. This combination therapy approach has the potential to combat biofilms and reduce bacterial virulence effectively. By synergistically targeting different aspects of biofilm formation and bacterial survival, the combination of phage-derived PSDs with other agents may provide an enhanced efficacy in treating biofilm-related infections. Further research and clinical studies are warranted to explore and validate the potential of this approach [75].

## 6. Antimicrobial Peptides and Peptidomimetics

Almost all organisms can produce various antimicrobial peptides (AMPs), also known as host-defense peptides (HDPs) [175,176]. In the Antimicrobial Peptides Database (https://aps.unmc.edu/, accessed on January 2023), 3569 antimicrobial peptides from six life kingdoms (from bacteria to animals and plants) were registered, including natural AMPs and synthetic peptides derived from natural AMPs. This database is constantly updated; for example, 177 new AMPs were registered in 2022. The HDPs are a class of small peptides (typically composed of 12 to 45 amino acids) that are part of the innate immune response with a broad spectrum of antibacterial, antiviral, antifungal, antiparasitic and antitumor activity [177,178].

Antibacterial peptides are a diverse group of compounds, produced both in a constitutive and induced way, with the common mechanism of action focusing on the interaction with the bacterial cell membranes, which is essential for their survival [175,179]. These peptides typically have cationic properties due to the abundance of lysine and arginine residues and amphipathic structures. Such structures enable AMPs to attach to hydrophobic lipid components and hydrophilic phospholipid groups effectively. The interaction of AMPs with the negatively-charged bacterial membrane is highly specific, while its amphipathic secondary structures enable their insertion into the lipid bilayer, disrupting the membrane’s integrity and permeability. This, in turn, leads to leakage of the intracellular contents, the loss of membrane potential, and, ultimately, bacterial cell death [179].

AMPs can exert a broad spectrum of activity, enabling them to effectively combat a wide range of bacterial species, including both Gram-positive and Gram-negative bacteria. This versatility can be attributed, in part, to the shared characteristics of cell membranes across different types of bacteria. While there may be variations in the membrane composition and structure among bacterial species, they generally consist of a lipid bilayer, which serves as a common target for AMPs [175,176,177,178,179].

AMPs are classified into two categories based on their mode of action: “membrane-acting peptides” and “non-membrane-acting or intracellularly-acting peptides” [180]. The membrane-acting peptides destabilize bacterial membranes, leading to their disruption. The exact mechanism of action is unknown, but three models are proposed. In the barrel stave model, amphipathic antimicrobial peptides penetrate the membrane and form channels allowing the outflow of the cytoplasm contents. In the toroidal pore model, peptide insertion into the membrane generates pore formation in which lipids intercalate between peptide helices. In the carpet model, peptides do not insert into the membrane but cover the membrane surface, and the loss of membrane integrity occurs via a detergent-like effect [175,178].

Apart from the destabilization of lipid bilayers, AMPs can modify the function of OM proteins (OMPs). OMPs, which constitute approximately one-third of all proteins in a bacterial cell, play crucial roles in various cellular functions such as the active transport of nutrients, respiration, the generation of proton motive force, ATP production, and intercellular communication. Therefore, even in cases where complete cell lysis does not occur, AMPs may exert a rapid bactericidal effect [181].

However, some of the bactericidal effects of AMPs are not solely attributed to membrane permeabilization or OMPs disturbance. Some non-membrane-acting AMPs have the ability to traverse across membranes without causing damage, yet they still disrupt normal cellular functions. These peptides can act independently or synergistically with membrane permeabilization, affecting essential biological processes such as replication [182,183,184], protein synthesis [185,186], or cell wall synthesis [178]. For example, the antimicrobial peptide buforin II (derived from natural buforin I, isolated from the stomach tissue of the Asian toad *Bufo bufo garagrizans*), inhibits the cellular functions of *E. coli* cells by binding to DNA and RNA after penetrating the cell membranes [187]. The structural differences between bacterial and eukaryotic membranes make the action of such peptides highly selective. Eukaryotic cell membranes have electrically-neutral, zwitterionic phospholipids on their surface, with which the positively-charged antibacterial peptides interact weakly. In addition, the presence of sterols (including cholesterol) in eukaryotic membranes stabilizes their structure, preventing the action of AMPs [188]. The membrane-targeting mechanisms of AMPs’ action determines not only their high toxicity to microorganisms but also reduces the possibility of the emergence of resistance to these agents. In addition, such a single antibacterial peptide is effective against a broad spectrum of microorganisms, including Gram-negative and Gram-positive microorganisms, with anti-biofilm and anti-persister activity [189].

Unfortunately, despite the advantages, the use of antibacterial peptides is limited due to their susceptibility to denaturation, proteolytic degradation, and instability in the bloodstream. In addition, their production in bacterial cells involves an expensive purification process [190]. Moreover, their limited selectivity against specific strains can result in the unintended killing or disruption of beneficial bacteria comprising the human microflora [175]. Consequently, applying AMPs as antimicrobial agents can disrupt the natural microbial balance in the body, leading to imbalances and potential health complications. AMPs’ properties, therefore, have been used to design compounds that mimic their features but are more stable, cheaper to obtain, and more specific in their action. AMPs derivatives include classical peptidomimetics, cationic polymers, and small molecules [179,191]. Mimetics retain the stereochemical properties and biological activity of the parent molecule; simultaneously, they possess a higher metabolic stability, membrane permeability, and selectivity at lower production costs [175,177,179].

Human, naturally-produced AMPs are found on human skin, in ears, and in urinary and respiratory tracts. Many of them, such as Casein 201, are a product of the enzymatic hydrolysis of commonly-occurring proteins. Human AMPs are classified into two principal families: cathelicidins and defensins. Cathelicidins are generally small cationic peptides with proteolytical activity; in humans, the only one is LL-37, which is the product of hCAP-18 processing [192]. Defensins are small, cysteine-rich cationic peptides expressed in neutrophils and epithelial cells. A characteristic feature of human defensins is the presence of β-sheet core structures, which are stabilized by disulfide bonds. The topology of this bond determines their division into three subfamilies (i.e., α, β, and θ; in humans, only α- and β-defensins exist). It was documented that defensins act directly on the membrane of bacteria, viruses, and fungi (for example, killing bacteria and inhibiting cell wall synthesis or inactivating virus replication), but also as immunomodulators important for innate immune responses to infections [193]. A synthetic non-peptide defensin mimic drug, brilacidin (PMX30063), exhibited broad-spectrum inhibitory activity against clinically-relevant Gram-positive and Gram-negative bacteria, and recently, phase 2 clinical trials for its therapeutic use in acute bacterial skin and skin structure infections was completed [194].

Antimicrobial peptides, called bacteriocin, are also produced by various bacterial species, specifically lactic acid bacteria, and show antimicrobial activities against various microorganisms such as bacteria and fungi. They are genetically encoded factors and do not have a toxic effect on eukaryotic cells. One of the best-known is nisin, produced by *Lactococcus lactis*. Nisin belongs to the group of lantibiotics (lanthionine-containing peptide antibiotics)—polypeptide antibiotics that contain the unusual sulfur-amino acids lanthionine and β-methyl-lanthionine. This bacteriocin exhibits antibacterial activity against a wide range of Gram-positive bacteria; Gram-negative bacteria are not susceptible to nisin due to the impermeability of the OM [195]. The mechanism of nisin action is associated with its interaction with lipid II and the blocking of peptidoglycan synthesis. For over 40 years, nisin has been used as a preservative in food production. There are also other bacteriocins with an effect similar to antibiotics, such as garvicine A (inhibiting septum formation) or microcin (blocking transcription and replication in target cells) [196].

As with traditional antibiotics, resistance to AMPs is also observed, although it is much less common [197]. Many pathogenic strains show a reduced susceptibility to AMPs based on constitutive and inducible resistance. For example, the major human antibacterial peptide LL-37 is degraded and inactivated by proteinases produced by the significant human pathogens *P. aeruginosa* (elastase), *E. faecalis* (gelatinase), *Proteus mirabilis* (metalloproteinase) and *Streptococcus pyogenes* (cysteine proteinase) [198]. Gram-positive bacteria have developed several strategies to prevent the binding of AMPs to the cell membrane, such as D-alanylation of lipoteichoic acids, the addition of L-lysine to the phosphatidylglycerol (lysinylation), the glycosylation of the wall teichoic acids or the deacetylation of the N-acetylmuramic acid [199]. Moreover, it has been shown that in Gram-negative bacteria, exposition to AMPs activates the PhoP-PhoQ (PhoPQ) system, which is responsible for remodeling the bacterial cell surface by modifying LPS molecules with aminoarabinose. Such a modification decreases the LPS net negative charge [200]. In a biofilm, alginate, an anionic extracellular polysaccharide, acts as a diffusion barrier to positively-charged antimicrobial peptides [201].

### Efficacy of AMPs against Persistent Bacteria and Biofilm

Despite the differences in structures and sequences between various AMPs, the bactericidal effect of most of them is associated with membrane damage; thus, they are also effective against metabolically-inactive cells and seem to be a promising agent against persister cells and biofilm formation [178,196].

De Breij et al. developed a panel of synthetic peptides derived from principal human cathelicidin LL-37 [202]. They found that one of them, SAAP-148 (a synthetic antimicrobial and antibiofilm peptide), killed multidrug-resistant *S aureus* and *A. baumannii*, prevented biofilm formation, decreased established biofilms biomass and eliminated persister cells. Such high SAAP-148 bactericidal activity, without resistance selection, has also been demonstrated during the treatment of skin wound infections in mice. Liu et al. used *B. subtilis* persisters to investigate their susceptibility to two synthetic cationic AMPs: SAAP-148 and TC-19 (derived from human thrombocidin-1) [203]. They reported that SAAP-148 and TC-19 rapidly killed *B. subtillis* vegetative and persister cells, causing an increased membrane permeability and changes in membrane fluidity; however, dormant *B. subtilis* spores were invulnerable to both the tested AMPs. It has been shown that another thrombocidin-based TC84 peptide, as well as the BP2 peptide (based on the LPS-binding domains of a bactericidal permeability-increasing protein), perturbed the inner membrane of *B. subtillis* germinated spores and, thus, affected the spore burst and the generation time [204].

Other studies have reported that short antimicrobial peptides rich in arginine ^®^ and tryptophan (W), containing varying numbers of sequence repeats, (RW)_n_-NH_2_, can inhibit the planktonic cell growth and biofilm formation of the *E. coli* HM22 strain, a hyper-persister producer, in a concentration-dependent manner [201]. Among the tested AMPs, (RW)_4_-NH_2_ showed the best killing efficacy and led to the killing of more than 99.7% of viable persister cells in a planktonic culture. Hou et al. demonstrated that these peptides can penetrate the EPS matrix and induce the dispersion of biofilm formed by the *E. coli* RP437 strain [205]. Such detached biofilm cells were effectively killed by octameric (RW) peptides and stayed susceptible to ofloxacin [201].

Applying AMPs with conventional antibiotics or using AMPs cocktails are also promising anti-biofilm and anti-persister strategies [206]. For example, melittin, the main component of bee venom, in combination with a broad spectrum of antibiotics (e.g., gentamicin, ciprofloxacin, vancomycin, and rifampin), is strongly effective against biofilm-forming MDR-MRSA and MDR-*P. aeruginosa* [207]. Rishi et al. have demonstrated that the nisin–ampicillin combination (0.5 μg/mL and 100 μg/mL, respectively) in the presence of mannitol (25 mM) eliminated *Salmonella* persister cells [208].

Due to their unique features (e.g., a broad spectrum of activity, low risk of resistance, and an ease of modification and synthesis of mimetics [180,181]), antimicrobial peptides could represent good candidates as next-generation antibiotics, primarily since they can be dedicated to combating dormant bacteria and biofilm at all stages of its formation.

## 7. Concluding Remarks and Future Perspectives

The emergence of antimicrobial resistance has posed a significant threat to public health worldwide. While traditional antibiotics primarily target essential cellular processes and macromolecules, persister cells have developed the ability to temporarily inactivate these targets, allowing them to survive antibiotic treatment. To effectively combat persister cells, alternative strategies are needed to directly kill these metabolically-dormant cells without relying on active cellular processes.

By disrupting membranes and the biofilm matrix and by inducing bacterial cell hydrolysis, we can develop a promising strategy against persistent bacteria. These targeted approaches not only overcome bacterial persistence but also enhance the effectiveness of existing antibiotics by increasing bacterial cell susceptibility. Disrupting the biofilm matrix enables a better penetration of antimicrobial agents to reach embedded bacterial cells. Simultaneously, the destabilization and destruction of the OM and cell wall heighten the permeability of bacterial cell membranes, facilitating the entry of antimicrobial agents and improving their efficacy.

Phage-derived lysins have emerged as promising antibacterial agents with tremendous potential. These proteins exhibit specificity towards bacteria, sparing the host microbiome, and they can effectively kill bacteria regardless of their metabolic activity. While they have shown a remarkable efficacy against Gram-positive bacteria and demonstrated the ability to eradicate bacteria in biofilms, there is a need to improve their effectiveness against Gram-negative bacteria and biofilm structures.

Phage-derived depolymerases, on the other hand, have shown effectiveness in disrupting the biofilm matrix; however, their use alone may not be sufficient to eliminate pathogenic bacteria from treated environments completely. Combining depolymerases with phage-derived lysins enhances their efficacy and leads to more successful outcomes [172].

Antimicrobial peptides have demonstrated exceptional potential in disrupting bacterial membranes and effectively targeting Gram-negative bacteria. They have also shown efficacy in disrupting preformed biofilms. Notably, AMPs can effectively target cell wall-deficient bacteria, such as L-forms and spheroplasts, where lysins may not be as effective. By employing AMPs alongside lysins and peptidoglycan hydrolases, we can harness the complementary actions of these agents and broaden our approach against persistent bacterial infections.

The construction of engineered chimeric (chimeolysins) or artificial (artilysins) lysins incorporating active components or domains derived from AMPs and PSDs-like molecules [113], or the simultaneous use of a combination of PGHs, PSDs [172], and/or AMPs, along with antibiotics, chemicals or other natural compounds, holds the greatest potential to inhibit and disperse the biofilms formed by both Gram-positive and Gram-negative bacteria. This comprehensive approach can effectively target both dormant and actively-dividing cells, as well as bacteria with intact cell walls or cell wall deficiencies. By simultaneously targeting membrane disruption, biofilm matrix disruption, and bacterial cell hydrolysis, we can strengthen our arsenal against persistent bacterial infections and reduce the emergence of resistant mutants. This multi-faceted approach provides promising treatment options for bacterial infections; however, further research and development efforts and interdisciplinary collaborations are essential to optimize these strategies, to gain a deeper understanding of their mechanisms of action, and to translate them into clinically relevant therapies.

## Figures and Tables

**Table 1:** The effectiveness of native and chimeric endolysins against antibiotic-selected persister bacteria and biofilm.

Name and Origin	Target	Planktonic Cells	Biofilm	Persister Cells	Ref.
**LysH5** native endolysin from *S. aureus* phage vB_SauS-phiIPLA88.	*S. aureus* *S. epidermidis*	MIC (strain dependent) 0.05–0.1 µM. Killing assay: 10× MIC (0.5 µM) of LysH5/for 3 h/37 °C. No viable cells detected. Re-growth: Minimal re-growth was detected *under conditions:* 10× MIC (0.5 µM) of LysH5/for 24 h/37 °C. Cells remained sensitive to LysH5 and were effectively killed with second dose of LysH5 10× MIC (0.5 µM) of LysH5/for 3 h/37 °C. Sub-MIC concentration: 0.25× MIC or 0.5× MIC. Bacterial growth was not inhibited. No induction of persister cells.	Disrupting assay: 24 h-old biofilm, 3× MIC (0.15 µM) of LysH5/for 6 h/37 °C. Destruction of a matrix structure. No viable cells detected. Inhibition in biofilm formation: Formation of biofilm is fully inhibited Even in the presence of sub-MIC concentrations of LysH5 (0.1 µM) Sub-MIC concentration: 0.25× MIC or 0.5× MIC Bacterial growth was not inhibited No induction of biofilm formation	Starting population: 10^8^ CFU. Persister cells isolation: treatment with 100× MIC of rifampicin (2 µg/mL) or 10× MIC of ciprofloxacin (3 µg/mL) for 4 h/37 °C. Persister population: 10^3^CFU. Killing assay: 10× MIC (0.5 µM) of LysH5/for 3 h/37 °C. No viable cells detected.	[105]
**P128** chimeric ectolysin created by the fusion of Lys16 ectolysin from *Staphylococcus* phage K, and SH3b lysostaphin CBD from *S. simultans* [123].	MRSA, MSSA and CoNS strains of *S. epidermidis* *S. haemolyticus* *S. lugdunensis*	MIC (strain dependent) 0.017–4.64 µM. Killing assay: 1× MIC of P128/for 1 h/37 °C, 2- to 3-log CFU reduction. 1× MIC of P128/for 2–4 h/37 °C, >4-log CFU reduction. Re-growth: 10× MIC (0.5 µM) of LysH5/for 24 h/37 °C. Minimal re-growth was detected. Cells remained sensitive to P128 and were effectively killed with second dose of P128.	Disrupting assay: 24 h-old biofilm, 1× MIC of P128/for 2 h/37 °C. Destruction of a matrix structure. No viable cells detected. P128 was able to eradicate the biofilm mass from the surface of microtiter plates and catheters with equal efficiency.	Starting population: 10^8^ CFU. Persister cells isolation: treatment with 50× MIC of vancomycin or 100× MIC of daptomycin for 6 h/37 °C. Persister population: 10^3^ to 10^5^ CFU. Killing assay: 4× MIC of P128/for 1 h/37 °C. No viable cells detected.	[117]
**Art-175** chimeric endolysin created by fusion of the sheep myeloid antimicrobial peptide of 29 amino acids (SMAP-29) and N-terminus of endolysin KZ144 [124].	MDR and laboratory strains of *P. aeruginosa*.	MIC 10 µg/mL. 2 µg/mL (with the addition of 500 µM EDTA). Killing assay: 25× MIC of Art-175 (+0.5 mM EDTA)/for 30 h/37 °C, >4-log CFU reduction. Time-laps microscopy: Cells from mid-exponential phase (OD_600_ 0.6), concentrated five times, 25× MIC of Art-175 (0.1 mg/mL). Complete lysis and dispersion of cellular debris after 6 min.	No data.	Starting population: overnight culture. Persister cells isolation: treatment with 5× MIC of ofloxacin for 5 h/37 °C. Persister population: 10^5^ CFU. Killing assay: 10× MIC Art-175 (40 µg/mL) (+0.5 mM EDTA)/for 1 h/37 °C, >5-log CFU reduction. No viable cells detected.	[97]
	MDR and laboratory strains of *A. baumannii*.	MIC (strain dependent) from 4 to 20 µg/mL or from 4 to 10 µg/mL (with the addition of 500 µM EDTA). Killing assay: 1× MIC of Art-175/for 1 h/37 °C. No viable cells detected. Time-laps microscopy: Cells from mid-exponential phase (OD_600_ 0.6), concentrated five times, Art-175 (0.4 mg/mL) (+0.5 mM EDTA). Complete lysis and dispersion of cellular debris after 3 s.	No data.	Starting population: overnight culture. Persister cells isolation: treatment with 60× MIC of tobramycin for 5 h/37 °C. Persister population: 10^3^ CFU. Killing assay: 30× MIC Art-175 (120 µg/mL) (+0.5 mM EDTA)/for 1 h/37 °C, >5-log CFU reduction No viable cells detected.	[69]
**CF-301** (PlySs2) native endolysin from prophage of *Streptococcus suis* genome [125].	MSSA and MRSA strains of *S. aureus* *S. epidermidis*.	Killing assay: 0.32 µg/mL of CF-301/for 24 h/37 °C. No viable cells detected. Re-growth: *Minimal re-*growth was detected *under conditions*: 0.032 µg/mL of CF-301/for 24 h/37 °C. Cells remained sensitive to CF-301 and were effectively killed with second dose of CF-301, 0.32 µg/mL of CF-301/for 24 h/37 °C. Sub-MIC concentration: 0.032 µg/mL. Bacterial growth slightly inhibited. No induction of persister cells.	MBEC (strain dependent) 0.125–0.25 µg/mL. Disrupting assay: 24 h-old biofilm: 32 µg/mL of CF-301/for 2 h/37 °C. 2-week-old biofilm: 32 µg/mL of CF-301/for 4 h/37 °C. Destruction of a matrix structure >5-log drop of viable cells after treatment. Inhibition in biofilm formation: formation of biofilm is fully inhibited at concentration down to 0.032 µg/mL. Mix-species biofilm: complete disruption of biofilm at 0.032 µg/mL. MIC of CF-301/for 24 h/37 °C. CF-301 was able to eradicate the biofilm mass from the surface of microtiter plates and catheters with equal efficiency.	Starting population: 10^8–^10^9^ CFU. Persister cells isolation: treatment with 100× MIC of daptomycin (100 µg/mL) or 3× MIC of ciprofloxacin (3 µg/mL) for 4 h/37 °C. Persister population: ~10^7^CFU for daptomycin, ~10^6^CFU for ciprofloxacin. Killing assay: 5× MIC (160 µg/mL) of CF-301/for 1–2 h/37 °C. No viable cells detected.	[126]

Abbreviations: (MIC) Minimal inhibitory concentration, (MBEC) Minimum biofilm-eradicating concentration, (CFU) Colony forming unit per ml.

## Data Availability

Not applicable.
